# Awareness of celiac disease among the public in Kuwait: a cross-sectional survey

**DOI:** 10.1186/s13104-023-06415-x

**Published:** 2023-07-03

**Authors:** Wafaa A. Qasem, Ali Al Roumi, Khaled Al Mojil, Hind Sakijha, Nouf AlMughamis

**Affiliations:** 1 Ministry of health of Kuwait, Al Kuwayt, Kuwait; 2 Kuwait University Medical School, Safat, 13110 Kuwait; 3 Wazin Telehealth, Kuwait City, Al Asimah, Kuwait

**Keywords:** Celiac disease, Health literacy, Knowledge, Kuwait, Gluten sensitivity

## Abstract

**Objective:**

Health literacy levels among the general population predict better health outcomes and uptake of health services. Inequities in health literacy and uptake of health services are often observed in deprived neighborhoods. There is a paucity of data on literacy regarding celiac disease in Kuwait. Therefore, the present survey aims to address this paucity of data.

**Results:**

We conducted a survey of 350 respondents in six governates of Kuwait. Although around 51% of respondents were aware of peanut allergy and gluten sensitivity, less than 15% were aware of celiac disease. More than 40% of respondents reported that a gluten-free diet should be promoted for everyone. Better awareness regarding CD was associated with Kuwaiti nationality, higher education levels and higher age. Among different governates, residents of Al-Asimah reported the highest awareness levels, while the rest of the governates did not differ significantly. While eating behavior did not significantly predict awareness regarding CD.

**Supplementary Information:**

The online version contains supplementary material available at 10.1186/s13104-023-06415-x.

## Introduction

Celiac disease (CD) is a chronic autoimmune condition that results from gluten sensitivity in genetically predisposed individuals [1]. With an estimated global seroprevalence of 1.4%, CD ranges from 1.1% in Africa to 1.8% in Asia [2]. It is histologically characterized by villous atrophy in the small intestine and presents with gastrointestinal symptoms like diarrhea, abdominal pain, and weight loss, as well as extraintestinal manifestations like anemia, arthritis, and dermatitis herpetiformis [1]. CD often coexists with other autoimmune conditions, such as type 1 diabetes, thyroid disease, and psoriasis [3], and its chronic nature leads to high levels of distress, anxiety, poor functioning, and reduced quality of life [4].

Given the significant impact of CD on individuals’ lives, it is crucial to increase public awareness and education about this condition. Enhancing knowledge of CD not only helps uncover its true epidemiological burden across different communities [5], but also plays a vital role in early detection, intervention, and management. With a better understanding of CD, the general public can recognize the symptoms more effectively, seek timely medical advice, and adhere to a gluten-free diet, which is the primary treatment for the condition.

Assessing public awareness and attitudes is crucial for effectively allocating resources and designing and implementing targeted education campaigns, screening, prevention, and treatment strategies [6]. Furthermore, higher health literacy levels generally correlate with better health outcomes [7]. Public education initiatives can include awareness campaigns, collaboration with patient advocacy groups, dissemination of information through various media channels, and incorporating CD education into school curricula and healthcare provider training.

While the Western world has seen a marked increase in CD incidence from the 20th to the 21st century [5], partly due to increased awareness, most epidemiological evidence stems from high-income Western populations [5, 6]. El-Metwally et al. highlighted the lack of CD research in Arab countries in their systematic review [8]. To date, no epidemiological studies on CD have been conducted in Kuwait. Al-Qabandi et al. reported a 47% frequency of CD in a small cohort of Kuwaiti children, most of whom were asymptomatic and identified through screening [9]. They suggest this represents only a fraction of the true prevalence, emphasizing the need to improve public knowledge and attitudes to enhance screening and diagnostic service uptake.

There is a scarcity of CD research in the Eastern world, highlighting the need for more studies to determine disease burden and public knowledge and attitudes to enable efficient resource allocation. Additionally, CD prevalence in Kuwait and other Arabian Gulf countries is underreported, despite potentially being similar to or higher than Western countries [6]. In response, we initiated a multiphase project (Celiac Disease in Kuwait: CeliaK) to assess knowledge, awareness, and practices among various population groups, including the general public, medical students, practitioners, allied health specialists, people with CD, and chefs and bakers. This study, the first in the series, aims to examine public awareness of CD and gluten sensitivity in Kuwait and underscores the importance of educating the public on this condition. The objectives are as follows:

1. Gauge awareness of CD among the general public in Kuwait.
2. Identify levels of awareness regarding CD in different governates of Kuwait.
3. Identify predictors of CD awareness among the general public in Kuwait.

## Main text

### Methods

This cross-sectional study was conducted in the six governates of Kuwait from July 2021 to September 2021. Before the conduct of this study, ethical approval (1717/2021) was sought from the ethical review board of the Ministry of Health, Kuwait. Convenience sampling was used to recruit the study participants in supermarkets located in the six governates of Kuwait. The study participants were recruited by teams of medical students stationed in these supermarkets. A total of 385 members of the public were conveniently selected and invited to take part in the study by the team of medical students. We could not employ random sampling in this study due to the lack of resources.

The students conducted the interviews using an electronic questionnaire administered on Android tablets. All study participants signed the informed consent form. They were assured confidentiality and anonymity and that no individual-level findings will be reported. For inclusion in the study, we approached adult (≥ 18 years old) Kuwaiti citizens and non-Kuwaiti residents in the six governates of Kuwait. We excluded participants who were below 18 years of age. The minimum sample size required was estimated at 385 using the following parameters: population size (N = 3,618,435), confidence level (95%) and margin of error (5%). The sample size was calculated using the formula: n = N × x / ((N − 1) × E² + x) [10].

The questionnaire administered by the medical students comprised three sections. The first section asked about the demographics of the study participants including gender, nationality, residential area, education levels, and combined family income and occupation. The second section included the respondents’ preference for home or restaurant-cooked meals and frequency of eating outside home.

The third section included six questions regarding awareness of celiac disease were asked. The first four questions were recorded on a dichotomous (yes/no) scale. Internal consistency of the questionnaire was assessed using Cronbach’s alpha value considered acceptable at 0.60 [11]. The awareness questions were summed together where higher scores indicated lower awareness levels. Two multiple-choice questions were asked regarding the organ most susceptible to gluten sensitivity and food sources of gluten. The questionnaire used in this suvery is presented as supplementary file 1.

All data were analyzed using Statistical Package for the Social Sciences (SPSSv. 27). Quantitative data were presented as mean (SD) and categorical data as frequencies (%). Multiple linear regression analysis was run to analyze predictors of awareness of CD. The covariates introduced in the multiple regression analysis were selected based on previous literature on awareness of CD in neighbouring countries [12, 13].

Before running linear regression, several analyses were run to ensure the assumptions regarding normality, linearity, homoscedasticity of residuals and multicollinearity were met. Histogram with superimposed normal curve were used to check homoscedasticity of residuals, Multicollinearity was assessed using values of Tolerance (TOL) and Variance Inflation Factor (VIF), considered acceptable at 0.25 and 4 respectively [14]. Statistical significant was defined as P ≤ 0.05.

## Results

Out of 385 respondents invited to participate in the survey, a total of 350 responded yielding a response rate of 90.90%. The mean age of study participants was 37.11 years (11.62). Nearly two-thirds of the participants were males (61.4%) followed by females (38.6%). A higher proportion of the participants were Kuwaiti (66%). A higher proportion of respondents were educated till high school (n = 152, 43.43%) or bachelor’s degree (n = 141, 40.3%). And only 22 (6.3%) of the participants worked in any healthcare field. Detailed demographics are presented in Table 1.

**Table 1:** Sociodemographic characteristics of 350 people, Kuwait, 2021

Characteristic	Frequency	
	n	(%)
**Age (years)**		
< 30	100	(28.6)
30–59	239	(68.3)
> 60	11	(3.1)
**Gender**		
Male	215	(61.4)
Female	135	(38.6)
**Nationality**		
Kuwaiti	231	(66)
Non-Kuwaiti	119	(34)
**Governorate**		
Al-Ahmadi	97	(27.7)
Al-Asimah	49	(14)
Al-Farwaniyah	78	(22.3)
Al-Jahra	41	(11.7)
Hawalli	67	(19.1)
Mubarak AlKabeer	18	(5.1)
**Level of education**		
Elementary/Middle school	32	(9.2)
High School or Diploma	152	(43.43)
Bachelor’s degree	141	(40.3)
Higher than bachelor’s degree (Master’s or PhD)	25	(7.2)
**Do you work in any of the medical fields?**		
Yes	22	(6.3%)
No	328	(93.7%)

The questionnaire on CD awareness yielded an acceptable internal consistency (alpha = 0.63). Among the respondents (Table 2), more than half of the participants (51.4%) had heard about peanut allergy and gluten sensitivity (51.14%). Only 14.9% of the respondents had heard of celiac disease. Aroung 43% responded that gluten-free diet is healthy for everyone irrespective of their health needs. Only 12.9% respondents correctly pointed out the source of gluten. And only 15.1% correctly identified that small intestine was the most susceptible organ to gluten sensitivity.

**Table 2:** Response to frequency of eating pattern and awareness regarding celiac disease

Statement	Response	Frequency	Percentage
**Eating pattern**			
How frequently (per week) do you eat at home?	Never	12	3.4%
	1 to 3 times	16	4.6%
	3 to 5 times	31	8.9%
	4 Daily	291	83.1%
How frequently (per week) do you eat at your relatives and friends’ house?	Never	91	26.0%
	1 to 3 times	215	61.4%
	3 to 5 times	22	6.3%
	Daily	22	6.3%
How frequently (per week) do you eat at restaurants and cafes?	Never	99	28.3%
	1 to 3 times	189	54.0%
	3 to 5 times	42	12.0%
	Daily	20	5.7%
**Awareness regarding Celiac diseas**			
Have you heard of peanut allergy?	Yes	180	51.4%
	No	170	48.6%
Have you heard of Celiac disease?	Yes	52	14.9%
	No	298	85.1%
Have you heard of gluten sensitivity?	Yes	179	51.1%
	No	171	48.9%
Do you think that a gluten free diet is healthy for everyone?	Yes	151	43.1%
	No	199	56.9%
Gluten is found in?	Potato flour, whole grains, and rice flour	73	20.9%
	Wheat and products with wheat (pie, cake)	107	30.6%
	All whole grains and products with whole grains (pie, cake)	45	12.9%
	Wheat, bulgur, oat, barley and products having them	24	6.9%
	I don’t know	101	28.9%
The most susceptible organ to gluten sensitivity is?	Stomach	134	38.3%
	Small Intestine	53	15.1%
	Liver	24	6.9%
	Skin	76	21.7%
	I don’t know	63	18.0%

Our analyses indicated that all assumptions for multiple regression analysis were met. Multiple linear regression yielded a statistically significant model (F = 10.76, p < 0.001), explaining 29.4% of the variance in awareness regarding CD. Better awareness regarding CD was associated with females (B=-0.21, 95% CI: -0.41 to -0.01), Kuwaiti nationality (B = 0.35, 95% CI: 0.12 to 0.59), higher education levels (B= -0.13, 95% CI: -1.82 to -0.83) and higher age (B=-0.01, 95% CI: -0.02 to -0.003). Among different governates, residents of Al-Asimah reported the highest awareness levels (B= -0.69, 95% CI: -1.02 to -0.38), while the rest of the governates did not differ significantly. While frequency of eating at home, relatives’ house or at restaurants did not significantly predict awareness regarding CD (Table 3).

**Table 3:** Predictors of awareness of Celiac disease (n = 350)

Variable	Unstandardized Coefficients		P	95.0% Confidence Interval for B	
	B	Std. Error		Lower Bound	Upper Bound
(Constant)	5.329	0.879	< 0.001	3.599	7.059
Age	− 0.012	0.005	0.009	− 0.021	− 0.003
Gender					
Male	Reference				
Female	− 0.213	0.101	0.036	− 0.412	− 0.014
Nationality					
Kuwaiti	Reference				
Non-Kuwaiti	0.353	0.120	0.004	0.116	0.589
Education	-1.329	0.252	< 0.001	-1.824	− 0.833
Frequency of eating at home	0.252	0.421	0.550	− 0.576	1.081
Frequency of eating at relatives’ houses	0.101	0.336	0.764	− 0.559	0.761
Frequency of eating at restaurants	− 0.519	0.308	0.093	-1.125	0.087
Do you work at any of the medical fields?					
No	Reference				
Yes	0.983	0.205	< 0.001	0.581	1.386
Governates					
Al-Ahmadi	Reference				
Al-Asimah	− 0.699	0.161	< 0.001	-1.016	− 0.381
Al-Farawaniyah	− 0.131	0.144	0.367	− 0.415	0.154
Jahra	− 0.086	0.168	0.608	− 0.418	0.245
Hawalli	− 0.262	0.147	0.076	− 0.550	0.027
Al-Kabeer	− 0.228	0.232	0.326	− 0.684	0.228

## Discussion

To our knowledge, this is the first study to gauge the level of awareness regarding celiac disease among the general population in Kuwait. This study presents crucial insights to inform the design of more robust studies in future. Although over 50% of the population was aware of peanut allergy and gluten sensitivity, only 15% had heard about CD. Most of the population had limited knowledge of the food products containing gluten or the organs most susceptible to gluten sensitivity. Kuwaiti nationals reported better awareness levels than non-Kuwaitis. The disparity in awareness of CD was largely driven by educational attainment.

We found a significant disparity in the knowledge regarding CD among the Kuwaiti population. These awareness levels were significantly lower than those reported in studies from Saudi Arabia [13], Turkey [12] and the UK [15]. An estimated 44–48% of the populations in these countries were aware of CD compared to 15% in our survey sample [12, 13, 15]. This situation is further worsened by a large proportion (43%) of the study sample advocating gluten-free diet as a healthy option for the general population. The highest levels of awareness regarding CD were reported in the Al-Asima governate of Kuwait. These poor levels of knowledge highlight the need for health literacy interventions in Kuwait.

These health education and promotion initiatives can improve the knowledge of varying presentations of CD and improve the uptake of screening services, and timely diagnoses and treatment. The potential of health literacy in improving diagnostic rates has been demonstrated in the literature. It has been illustrated that the increased use of serological screening by primary health care workers can significantly improve the rates of diagnosis of CD, however, the application of such a widely available serological screening test remains not utilized [16]. Improved uptake of these serological screening services could help identify the hidden burden of CD, especially among asymptomatic individuals.

We found that better awareness regarding CD was associated with higher educational level attainment. This finding is corroborated by a recent study conducted in Turkey that found a positive association between education level and awareness of CD (p < 0.05) [12]. This association is also consistent among different occupation groups such as chefs where those with higher qualifications report better knowledge of CD (83%) than those with lower qualifications (52%) [17]. This finding has important policy implications. Firstly, it warrants the need for concentrating health awareness initiatives in neighborhoods with poorer educational attainment. This is because health literacy has been demonstrated as a factor that can help explain inequities in health outcomes [18]. Moreover, poor health literacy increases the risk of having difficulties navigating healthcare and treatment services [19].

Our analyses revealed several important sociocultural predictors of awareness regarding CD. Kuwaiti socio-culture environment is predominantly conservative [20]. Kuwaiti traditions value strong ties between family, neighbors, and friends. The knowledge regarding CD becomes important in family-centred environments where family get-togethers are encouraged. As noted in our results, 54 to 60% of Kuwaiti nationals reported eating at restaurants and relatives’ houses, at least 1 to 3 times a week. Moreover, females being the primary homemakers in the conservative society of Kuwait reported lower awareness levels regarding CD. Both these factors are important because the dietary needs of people with CD may not be tended to in Kuwait because of poor awareness regarding it.

This study has several strengths and limitations. It reports awareness regarding CD in a study sample recruited from six governates of Kuwait. The response rate was adequate at 91%. However, there are two main limitations of this study design. The survey respondents were sampled conveniently, which might limit the generalizability of these results to the entire Kuwaiti population. The cross-sectional nature of the study design limits inferences related to causality; therefore, the results of linear regression analysis should be interpreted with caution. Although the present utilized a short survey to gauge awareness regarding CD, we recommend future studies employ more robust and detailed questionnaires to gain more valuable insights.

In conclusion, the present study reveals important insights regarding knowledge of CD among the general population in Kuwait. Poor awareness and knowledge regarding CD were demonstrated among Kuwaiti expatriates and people with poor education. We recommend that health literacy interventions be provided to the general population to raise awareness of CD. These interventions should be prioritized for areas reporting poorer educational attainment.

## Electronic supplementary material

Below is the link to the electronic supplementary material.

Supplementary Material 1

## Data Availability

The dataset associated with this study can be made available on request from the corresponding author.

## Abbreviations

CD: Celiac disease
