# Supplementary material for: Awareness of celiac disease among the public in Kuwait: a cross-sectional survey
Source: BMC Res Notes. 2023 Jul 3;16:133. doi: 10.1186/s13104-023-06415-x (PMC10318624; doi:10.1186/s13104-023-06415-x)
Supplement: Supplementary file 1 — Supplementary Material 1 [file 13104_2023_6415_MOESM1_ESM.pdf]

Awareness of celiac disease among the public in Kuwait: A cross-sectional survey

Questionnaire

Participant characteristics

1. **Age:** _______________

2. **Gender**

◯ Male

◯ Female

3. **Nationality**

◯ Kuwaiti

◯ Expatriate

4. What governorate do you live in?

◯ Al-Ahmadi

◯ Al-Asimah

◯ Al-Farwaniyah

◯ Al-Jahra

◯ Hawalli

◯ Mubarak Al-Kabeer

5. What is the highest degree or level of education you have completed?

◯ Elementary/Middle school

◯ High school

◯ Diploma

◯ Bachelor’s degree

◯ Higher than bachelor’s degree (Master’s or PhD)

6. What is your combined family income?

◯ Less than 500 KD

◯ 500 to 1,000 KD

◯ 1,001 to 1,500 KD

◯ 1,501 to 2,000 KD

◯ More than 2,000 KD

7. Do you work at any of the medical fields?

◯ Yes

◯ No

Pattern of eating

8. How frequently (per week) do you eat at home?

◯ Never

◯ 1 to 3 times

◯ 3 to 5 times

◯ 4 Daily

9. How frequently (per week) do you eat at your relatives and friends house?

◯ Never

◯ 1 to 3 times

◯ 3 to 5 times

◯ Daily

10. How frequently (per week) do you eat at restaurants and cafes?

◯ Never

◯ 1 to 3 times

◯ 3 to 5 times

◯ Daily

Awareness regarding Celiac disease

11. Have you heard of peanut allergy?

◯ Yes

◯ No

12. Have you heard of Celiac disease?

◯ Yes

◯ No

13. Have you heard of gluten sensitivity?

◯ Yes

◯ No

14. Do you think that a gluten free diet is healthy for everyone?

◯ Yes

◯ No

15. Gluten is found in?

◯ Potato flour, whole grains, and rice flour

◯ Wheat and products with wheat (pie, cake)

◯ All whole grains and products with whole grains

◯ Wheat, bulgur, oat, barley, and products having them

◯ I don't know

16. The most susceptible organ to gluten sensitivity is?

◯ Stomach

◯ Small Intestine

◯ Liver

◯ Skin

◯ I don’t know
